# Rethinking entrenched narratives about protected areas and human wellbeing in the Global South

**DOI:** 10.14324/111.444/ucloe.000050

**Published:** 2022-11-16

**Authors:** Emily Woodhouse, Claire Bedelian, Paul Barnes, Gisella S. Cruz-Garcia, Neil Dawson, Nicole Gross-Camp, Katherine Homewood, Julia P.G. Jones, Adrian Martin, Elisa Morgera, Kate Schreckenberg

**Affiliations:** 1 Department of Anthropology, University College London, London, UK; 2 International Institute for Environment and Development, London, UK; 3 EDGE of Existence Programme, Zoological Society London, London, NW1 4RY, UK; 4 Sowing Diversity = Harvesting Security, Oxfam Novib, The Hague, The Netherlands; 5 School of International Development, University of East Anglia, Norwich, UK; 6 Commission on Environmental, Economic and Social Policy, International Union for the Conservation of Nature, Gland, Switzerland; 7 Boston College, Morrissey College of the Arts & Sciences, Environmental Studies Program, Chestnut Hill, MA, USA; 8 College of Environmental Sciences and Engineering, Bangor University, Bangor, UK; 9 Law School, University of Strathclyde, Glasgow, UK; 10 Geography Department, King’s College London, London, UK

**Keywords:** conservation, development, ecosystem services, equity, governance, poverty, protected areas, social justice wellbeing

## Abstract

Attempts to link human development and biodiversity conservation goals remain a constant feature of policy and practice related to protected areas (PAs). Underlying these approaches are narratives that simplify assumptions, shaping how interventions are designed and implemented. We examine evidence for five key narratives: 1) conservation is pro-poor; 2) poverty reduction benefits conservation; 3) compensation neutralises costs of conservation; 4) local participation is good for conservation; 5) secure tenure rights for local communities support effective conservation. Through a mixed-method synthesis combining a review of 100 peer-reviewed papers and 25 expert interviews, we examined if and how each narrative is supported or countered by the evidence. The first three narratives are particularly problematic. PAs can reduce material poverty, but exclusion brings substantial local costs to wellbeing, often felt by the poorest. Poverty reduction will not inevitably deliver on conservation goals and trade-offs are common. Compensation (for damage due to human wildlife conflict, or for opportunity costs), is rarely sufficient or commensurate with costs to wellbeing and experienced injustices. There is more support for narratives 4 and 5 on participation and secure tenure rights, highlighting the importance of redistributing power towards Indigenous Peoples and Local Communities in successful conservation. In light of the proposed expansion of PAs under the post-2020 Global Biodiversity Framework, we outline implications of our review for the enhancement and implementation of global targets in order to proactively integrate social equity into conservation and the accountability of conservation actors.

## Introduction

In 2010, state parties to the Convention on Biological Diversity (CBD) agreed to increase protected areas (PAs) to 17% of terrestrial and inland waters and 10% of marine and coastal areas [1]. Significant advances have been made towards this target [2]. Support is coalescing around a global target for the post-2020 Global Biodiversity Framework of 30% protection by 2030 [3] while the ‘Nature Needs Half’ campaign has also gained considerable momentum [4]. Recent studies reinforce the global importance of well-managed PAs in protecting species richness and abundance [5] and maintaining wildlife populations [6]. However, it has long been recognised that while they may contribute to wellbeing at the global scale through the ecosystem services (ES) they deliver such as carbon sequestration and hydrological functions [7], PAs can also bring costs as well as benefits to local populations [8]. This is especially true for the rural inhabitants of the Global South, who can experience opportunity costs [9], damage from wildlife [10] and displacement through eviction and cultural exclusion [11]. Protecting 50% of the Earth is likely to impact more than a billion people [12].

With the rise of the concept of sustainable development in the early 1980s and especially in the wake of the 5th World Parks Congress in 2003, the idea that conservation and development are interdependent became mainstream [13]. It is now well accepted that the global good of conservation should not be delivered in a way that harms local people, and should in fact respect and contribute to the realisation of human rights [14,15]. The Durban Accord developed at the 2005 World Park Congress goes further to state that PA management must strive to reduce, and in no way exacerbate, poverty [16]. CBD parties, in turn, have emphasised the need for PAs to be established and managed through equitable processes that recognise and respect the rights of indigenous peoples, local communities and vulnerable populations [1]. A suite of approaches such as ecotourism, compensation, alternative livelihood schemes, community-based natural resource management (CBNRM) and efforts to secure tenure rights aim to meet these commitments on the ground. Calls to decolonise conservation have become increasingly forceful in recent years, casting new light on debates around the rights of Indigenous Peoples and Local Communities, participatory processes, benefit-sharing, social justice and equity, not least through recognition of the neocolonial nature of many conservation interventions [17,18]. There is urgent need to identify conservation approaches most likely to strengthen synergies between social and ecological gains that encompass equity and justice.

Studies examining the relationship between PAs and human wellbeing paint a rather mixed picture of how policies have worked in practice. Controversy over PAs has partly been fuelled by the variety and distribution of impacts, the different methods used to capture them, and the different types of governance and management in place [19]. Reviews of the social impacts of PAs (e.g., [20,21]) have usefully characterised the types of outcomes evidenced, but have not fully examined the processes through which different outcomes arise for different social groups. A number of quantitative studies have shown a generally positive impact of PAs on economic wellbeing (e.g., [22]). While averaged material indicators allow analysis over larger scales, they miss valued aspects of human wellbeing and ignore questions of equity. Recent approaches to the social dimensions of PAs have taken a multi-dimensional view of human wellbeing that looks beyond material circumstances, to a subjective evaluation of one’s own life, and a relational component that focuses on how people engage with others to achieve their goals [23]. Conceptualisations of equity have also expanded from looking at the distributional impacts to encompass recognition of rights and values, and procedural aspects [24].

Despite sometimes polarised debate and contested evidence, attempts to link human development and conservation goals remain a constant feature of policy and practice related to PAs [13,25]. Underlying these approaches are stories or narratives that have persisted through time about the relationships between the wellbeing or actions of local communities and conservation outcomes. The power of such narratives lies in the way they simplify complex and uncertain situations, but can unhelpfully become ‘blueprints’ for interventions that are ineffective in particular contexts [26]. Simplified stories serve to make decision-making more manageable and stabilise assumptions, becoming embedded in funding structures and networks of power [27]. For example, in the case of Namibian conservancies, win–win narratives are important for ‘public showcasing of success’ by donors and non-governmental organisations (NGOs), making critique often unwelcome [28]. Acknowledging shortcomings and understanding complexities, however, is likely to ultimately improve the sustainability of interventions [29].

In this paper we examine evidence for five common narratives that underlie and justify PA establishment or management. The first narrative is that because the poor are most dependent on ES, conservation interventions that protect ecosystems will alleviate poverty, that is, they will be ‘pro-poor’ [30]. On the flip side, the assumption that poverty reduction will reduce people’s reliance on natural resources and therefore support conservation has underpinned popular integrated conservation and development projects (ICDPs) since the 1980s [31]. Where harm to local populations is unavoidable, the notion that this can be sufficiently compensated for through economic schemes, has had material consequences, for example, many millions of dollars being spent to offset the damage caused by wildlife around the world [32]. Participation by local communities is a mainstream idea in PA governance on the basis that it leads to more effective conservation than top-down approaches ([33]: although in practice ‘participation’ ranges from largely rhetorical to genuine engagement). Finally, secure tenure rights over land and resources for communities are increasingly considered an important foundation for attaining positive conservation outcomes [34]. The five narratives are defined in Box 1.

Box 1. Definitions of narratives

**N1. Conservation is pro-poor:** Because poor people are disproportionately dependent on ES, PAs that protect or enhance those services will alleviate poverty

**N2. Poverty reduction benefits conservation:** Because poor people are disproportionately dependent on ES, improving their material wellbeing will reduce pressure on PAs

**N3. Compensation neutralises costs of conservation:** Unavoidable costs of PAs for local people can be adequately offset by providing appropriate compensation

**N4. Participation is good for conservation:** Local participation in PA governance is a route to more effective conservation

**N5. Secure tenure rights for local communities support effective conservation:** Secure and well-defined rights of tenure to land and resources underpin positive social and ecological outcomes in and around PAs

The objective of this paper is to examine *if* and *how* each narrative is supported or countered by the evidence from low- and lower middle-income countries. We use a mixed-method synthesis combining a critical review of recent relevant peer-reviewed literature and expert key informant interviews. We aim to capture wellbeing and equity outcomes across social, economic and political dimensions. In the context of ambitious aims for expanding PAs, better understanding of the complex trade-offs and synergies across social and ecological outcomes will be vital in negotiating and managing how post-2020 targets are translated into governance structures and implemented on the ground. There is a growing recognition that conserved areas outside formally designated PAs, such as indigenous and community managed areas, and privately managed areas have a role to play in conservation [35]. In line with latest policy we encompass the full range of PAs [36], including these other conservation areas, in both terrestrial and marine systems.

## Methods

The narratives were identified during a 2-day workshop through deliberative processes based on participants’ (conservation researchers and practitioners) knowledge. This involved identifying possible narratives in small groups, then discussing their importance and popularity in forming the basis for PA policy and practice based on participants’ experience and with reference to international conservation policy documents. The narratives were subsequently validated through a review of the websites of 169 conservation organisations operating in lower- and lower middle-income African countries and internationally (see Supporting Information, Conservation organisations; [37]) and through expert interviews (see below). One hundred and thirty-eight of these organisations employed at least one of the narratives in materials that described their work with more focus on N1 (118), N2 (108), N4 (84), than N3 (53) and N5 (39). Interviewees stated high levels of familiarity with the narratives especially N2, N4 and N5 (Supporting Information, Interview validation). We chose a mixed-methods approach to examine the complex relationships between PAs and human wellbeing within each narrative. We combined relevant elements of systematic reviews to select literature in a transparent and unbiased way [38] but limited the sample of papers in order to allow more depth of analysis, and carried out a narrative review more appropriate to capturing complexity, process and context [39,40]. On the principle that understanding complex conservation issues will benefit from a range of evidence from different sources [41], and recognising the value of expert knowledge and experience [42], we complemented the literature with key informant interviews with conservation researchers and practitioners.

### Literature search

**Figure 1:**
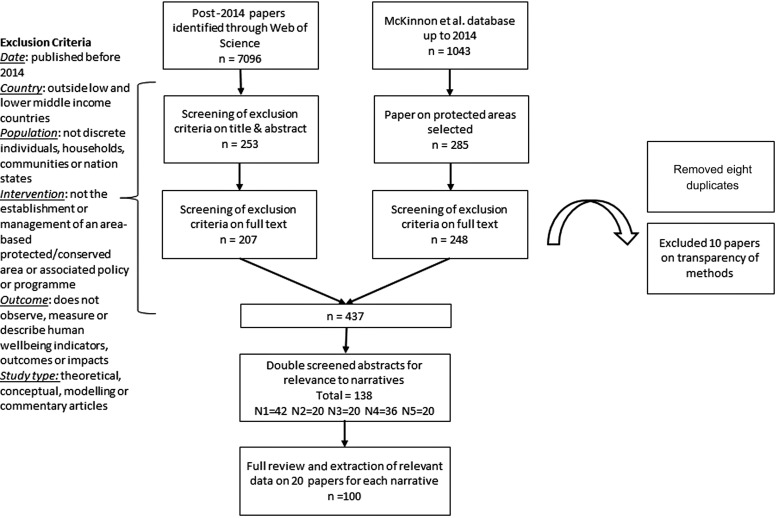
Screening process and number of articles at each stage.

To search the literature on the social outcomes of PAs we combined two databases of evidence. First, we used a systematic map and database of 1043 studies published up to 2014 by McKinnon et al. [43] (available at https://natureandpeopleevidence.org), on the linkages between conservation interventions and human wellbeing in terrestrial and marine systems. We selected only peer-reviewed articles related to ‘area protection’ and/or ‘area management’ interventions in low- and lower middle-income countries only as designated by the World Bank (Supporting Information, World Bank Economies). We selected articles published after 2006 with a study date after 2003, to capture recent studies more reflective of people-centred approaches to PA conservation after the Durban Accord (2003) and the Millennium Ecosystem Assessment [44]. Our search resulted in a set of 285 relevant articles. These were screened on full text based on our exclusion criteria, reducing the set to 248 articles (Fig. 1; Supporting Information, Exclusion criteria).

Second, we updated the database beyond 2014 with our own systematic literature search. We used the same search terms as McKinnon et al. [43], but limited the intervention search terms to those related to PAs and other area-based conservation measures, drawing upon terms used in a systematic review by Pullin et al. [20] of protected areas and supplementing these with our own. Using Web of Science, we limited the search to English language, peer-reviewed articles, published after 2014 (Supporting Information, Search terms). The search retrieved 7096 articles. These were imported into an EPPI-Reviewer 4 and screened based on our exclusion criteria, first on title and abstract, and second on full text, reducing the articles to 207. These were combined with the 248 articles identified from McKinnon et al. [43]. Duplicates were removed and 10 papers were excluded due to poor transparency of methods, resulting in a final set of 437 articles.

The 437 article abstracts (published between 2003 and 2017) were double screened for relevance to one or two narratives (with primary and secondary relevance agreed), resulting in 138 papers selected as relevant by two reviewers. Twenty papers were randomly selected from each set of papers per narrative for data extraction. Where fewer than 20 of the papers had primary relevance to one narrative, papers were selected from those that had secondary relevance. This was the case for N2 (one paper), N3 (one paper) and N5 (two papers). More papers were relevant to the Pro-poor (N1) and Participation (N4) narratives than to the remaining three narratives (Fig. 1). The location of PAs in the 100 papers were weighted towards Africa (63) and Asia (36), with only one paper from the Americas, and none from Oceania or Europe. This reflects both the disproportionate number of African and Asian countries categorised as low- and low middle-income (Supporting Information, World Bank economies), and publishing bias. Sixteen African countries and nine Asian countries are represented in the papers but with certain countries disproportionately represented: Tanzania (18); India (12); Nepal (10). Each paper was reviewed using a standard coding tool developed in Google Forms to extract and categorise the data relating to the study, PA, social outcomes and narratives (Supporting Information, Codebook).

### Expert interviews

We carried out a total of 25 semi-structured interviews (either in person or on video call), including eight with academic researchers working on projects funded by the Ecosystem Services for Poverty Alleviation (ESPA) programme [45] and 17 with contacts of the authors working outside of academia. Interviewees were selected with the aim of achieving representation from different types of organisations across the globe, including international and in-country NGOs, state agencies and research organisations (Supporting Information, Non-academic interviewees) and for their experience in the governance of PAs and/or understanding their impacts. Interviewees were asked about their familiarity with each of the narratives and experience of their validity (Supporting Information, Interview questions). Interviews captured expert knowledge, long-term field experience and supported the identification and interpretation of key themes across the narratives.

### Narrative synthesis

The publications that were randomly selected encompassed a range of designs, methods and data types (quantitative and qualitative), which was useful in exploring causal linkages, processes of change and contextual factors [46]. We assumed a level of quality through the peer-review processes of the journals, and used our expertise in the social sciences to assess the weight of evidence in support of the narratives in each paper which was categorised into strong (results fit the narrative with little deviation), partial (results are mixed or do not demonstrate the narrative in full) or none (results provide no support). Data from both the literature and interviews were combined in the analysis. A narrative synthesis aims to provide insight and deepen understanding rather than conventional systematic reviews which aim to answer specific questions [42]. We took a thematic synthesis approach [47], annotating and identifying themes within the extracted data and refining them in an iterative process. The findings are organised around these themes for each of the narratives in the text below and summarised in Table 1. The author carrying out the narrative review for each narrative reread the papers, extracted data, and interview transcripts, and the support categorisation and narrative text were discussed and agreed with the lead author.

**Table 1.** Summary of evidence on the narratives

Narrative shorthand	Summary of evidence for the narrative
N1: Conservation is pro-poor	PAs can contribute to basic human needs and material poverty alleviation, but this is dependent on access. Due to exclusion, the poor commonly experience costs from PAs. Where multiple dimensions of wellbeing are included in studies, there are trade-offs and complexities in outcomes.
N2: Poverty reduction benefits conservation	For improvements in wellbeing to benefit conservation, promoted changes must be suited to local values, linked to biodiversity and inclusive. Promotion of alternative livelihoods often leads to unintended negative social and ecological outcomes.
N3: Compensation neutralises costs of conservation	Material compensation is less relevant for supporting positive conservation outcomes than recognition of local social and cultural practices, and decision-making influence. Compensation schemes are also often hampered by low transparency and unequal impacts.
N4: Local participation is good for conservation	Meaningful participation, or more broadly the quality of governance, and extent of rights and control afforded to local communities, influence their motivation and capacity to conserve. Consultative participation or weak inclusion of marginalised groups hinders conservation.
N5: Secure tenure rights support effective conservation	Secure tenure rights can empower local communities to effectively conserve, but crucially this entails respect for customary and communal access systems. Conservation governance that only recognises formal property rights or causes tenure insecurity produces unequal impacts and weak local legitimacy.

## Results

### Narrative 1: Conservation is pro-poor

This narrative asserts that as it is the poorest people who are most dependent on ecosystems for their livelihoods, biodiversity conservation through PAs can alleviate material poverty by securing provisioning ES such as food and fuel, and regulating services such as clean water [48,49]. This narrative would suggest that when there is loss of access to extractive uses economic benefits can come through tourism or payment mechanisms, for example, wildlife management areas (WMAs) are assumed to reduce poverty through increased income revenues from wildlife [50].

**Figure 2:**
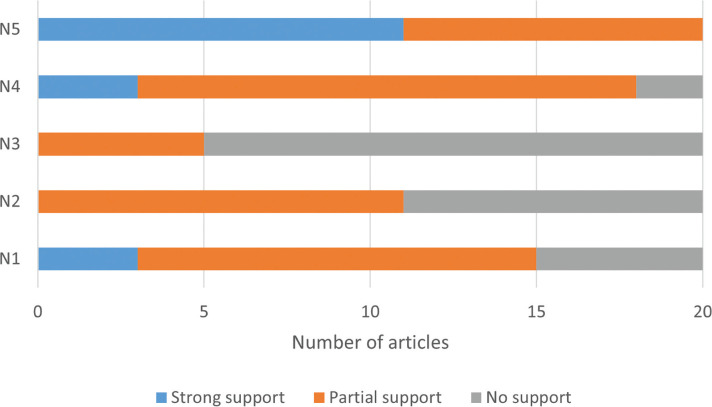
Level of support shown by articles for each narrative.

**Figure 3:**
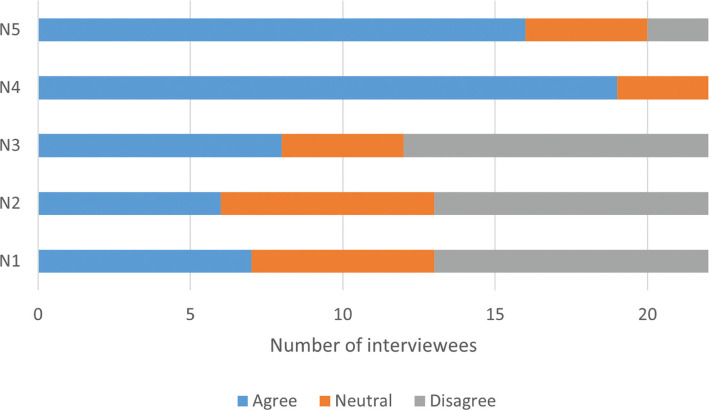
Interview responses on the validity of each narrative. n = 22 as three interviewees chose not to answer these closed ended questions in the interviews.

Of the 20 selected papers, three provided strong support for the narrative, with five showing no support or providing evidence against, and a further 12 showing some support but with mixed (positive and negative) or weak effects (Fig. 2). Interviewees were divided in their support (Fig. 3). The explanation for these divergent results rests on several factors. First, the extent that PAs are pro-poor centres on people’s access to ES and their benefits, in turn dependent on the management system which can range from strictly protected to community-managed areas. Although some services can benefit all across a landscape (e.g., flood protection), the negative impact of exclusion for other services was evident in our sampled papers in both terrestrial [51,52] and marine PAs [53]. The poor living in and around PAs are also more exposed to ecosystem ‘disservices’ from wildlife such as crop-raiding [52,54] which can have wide-ranging and hidden impacts such as on psychological health and education [55].

Nine of our interviewees questioned the logic of the narrative: the poor often do not benefit from ES from a PA, and in fact are more likely to lose out. The wealthy are better placed to benefit due to their higher capacity to capture resources and bypass access restrictions, especially if governance is weak. The papers that disaggregated data according to wealth supported this idea. For example, compared with poorer households, wealthy households participate more in Payment for Ecosystem Services (PES) schemes in PAs in Cambodia [56], benefit more in terms of food security from CBNRM in Tanzania [57] and access benefits from devolved forest management in Ethiopia [51]. Indigenous groups who are already socially marginalised are at particular risk of disproportionate harms if they are not given special protection, such as the Twa whose livelihoods and culture are intertwined with native forests in Rwanda [58]. The poorest and landless are more dependent on resources from PAs, and by necessity have to risk fines and imprisonment where there are legal restrictions [58,59]. Tourism benefits are also prone to elite capture without redistribution policies in place [55,56,60].

Where poor local residents are not excluded from the benefits of conservation, the papers showed limited evidence that PAs are a pathway out of poverty, a message reflected in the literature on linkages between ES and poverty alleviation [48,61]. PAs more readily act as a social safety net preventing further poverty. For example, those most reliant on income from the Chiradzulu Forest Reserve, Malawi, are among the poorest, who have little education, more dependents, fewer assets and are more likely to be women [62]. The provision of forest products to the poor from Kibale National Park, Uganda protects them against desperation sales of farmland and thus sinking deeper into poverty [63]. One paper in our sample showed neutral impacts on food security [64], and Canavire-Bacarreza and Hanauer [65] show an average reduction in poverty in municipalities in Bolivia that have at least 10% of their areas covered by PAs. These papers represent a growing body of robust quantitative research providing evidence that some PAs in the Global South can reduce poverty or at least do not necessarily increase it especially where there is tourism and or the PA is not strictly protected (e.g., [22,66,67]) but do not look beyond objectively measured average material poverty and health.

The papers in our sample that showed strong support for the narrative used variables and metrics centred on material wellbeing [62,63] with the exception of Canavire-Bacarreza and Hanauer [65], who measured average effects on a poverty index which incorporates education and health. Research that looked at changes in diverse aspects of wellbeing (e.g., non-use values, food security, empowerment) paints a more complex picture with gains in some variables and losses or no change in others [54,57,68]. Likewise, interviews suggested that the most important costs and benefits for wellbeing and local support for PAs may not be material, for example, cultural knowledge or a sense of autonomy. Gurney et al. [69] highlight this point: despite a positive impact on livelihood diversity and wealth from marine PAs in Indonesia, subjective wellbeing was negatively affected most likely due to increased conflict and unmet expectations.

To fully understand the impacts of PAs, consideration must be given to the wider spatial, temporal and socio-economic context. The effect of PAs may be relatively limited where there are strong drivers of poverty or development related to market access, land policy and population changes [52,56]. Dawson and Martin [58] highlight how positive outcomes for biodiversity and wellbeing are in part dependent on the governance of the wider landscape outside of PAs and therefore provision of alternative vital resources. Studies that investigate impacts at different scales show that the validity of the narrative can change through time and space with trade-offs involved. Those closest to PAs or in more accessible areas tend to access benefits derived from ES such as income [62] or tourism infrastructure [70], but are also exposed to the damage from wildlife [55]. Temporal dynamics affect how benefits are realised: for example, benefits may be felt most during implementation when funding is available [69], or conversely may take time to be realised [57]. Positive benefits from long-term sustainability involve time-lags and in the case of mangrove protection, counteracted immediate losses of resources but with uncertain trajectories [71].

Overall, our analysis suggests that it is possible for PAs to alleviate material poverty but the extent to which the PA will benefit the poor depends on a range of factors including restrictions to locally important ES (especially provisioning services), whether local people have the capability (related to wealth and status) to benefit from ES, and how the PA and wider landscape is governed.

### Narrative 2: Poverty reduction benefits conservation

The idea that resource overexploitation is a response to poverty was first popularised amongst conservationists in the World Conservation Strategy of 1980 [72] and since then has formed the basis for an instrumental argument that poverty alleviation should be integral to conservation initiatives. This narrative, to varying extents, underpins ICDPs, alternative livelihoods and revenue sharing schemes from ecotourism. There are two principal rationales for such programmes: first, to provide economic substitutes that reduce reliance on natural resources and lessen environmentally damaging behaviours; and second, to increase local acceptance and support for conservation, creating positive change in attitudes and behaviours [73].

There was mixed support for this narrative in our sampled literature and our interviewees were divided on its validity. Several papers did show how schemes designed to improve people’s material wellbeing positively influenced attitudes towards conservation (e.g., [74,75]), but this did not extend to strong evidence of change in behaviour or biodiversity outcomes. Those papers which studied behaviour showed some effects on reported extractive activities which were small and inconsistent [76] or reflected potential confounding factors [75]. Ecological outcomes were not maintained in the longer term [77] or were not clearly linked to social improvements [78]. The relationship between conservation attitudes and behaviour is not straightforward, and the evidence highlighted the need to understand not only attitudes towards conservation but towards PA staff and conservation organisations which can be instrumental in creating support [74].

The experience of our interviewees suggests that the narrative is more valid when people perceive a direct link between the PA and benefits, they receive. This linkage can be achieved in two main ways; first where the livelihood intervention is materially dependent on effective conservation (ecotourism, agro-forestry and resource access), and second where there are economic incentives such as the conditionality of PES payments. In fact, the PES concept emerged as a counter-narrative to the assumption that support for local incomes automatically enhances conservation effectiveness, instead arguing that such support needs to be conditional on conservation performance [79]. Our sample included seven papers which looked at interventions in the former category, but positive effects were not more strongly evidenced than in other livelihood schemes. A case study provided by an interviewee documents one positive example: in the Amani Butterfly Project in northern Tanzania, successful butterfly farming relies on the existence of the PA natural forest and income from butterfly farming was positively associated with participation in forest conservation [80]. Farmers perceive a link between butterfly farming income and forest conservation, thus motivating behaviours such as tree planting and reporting of illegal activities. Although having a more logical basis, our interviewees suggested that in reality the socio-economic conditions conducive to such an arrangement are rare. The literature also suggests that these projects are no less susceptible to failures in implementation such as administrative delays, lack of technical support and unequal distribution of benefits which can all lead to erosion of trust and cooperation [81,82]. Our two sampled papers on PES, show that conditionality provides a better guarantee of positive environmental outcomes but impacts on poverty are dependent on the magnitude of payments which can often be small, and there is a tendency for benefits to be captured by elites [83,84].

Providing benefits is not a guarantee of attitude and behaviour change. In many cases, especially where time is not a limiting factor, these livelihoods will supplement rather than substitute resource extraction. Where there are big risks associated with conservation such as human–wildlife conflict, these may be a barrier to changing attitudes even where people are benefiting [85]. On the other hand, where there are large economic gains from alternatives, they may have the unintended consequence of exacerbating pressure on PAs by encouraging in-migration or reinvestment [86,87]. Livelihood decisions are driven by a range of factors beyond economic costs and benefits. Projects implemented with little regard to local community needs or cultural identities which may be closely tied to resource-dependent livelihoods such as fishing are more likely to fail [88]. In marine PAs in the Philippines, where economic expectations are not being met, this has led to negative attitudes towards conservation. Chaigneau and Brown [89] suggest in this case that it is more realistic and sustainable to emphasise non-material bequest and aesthetic values which also produce positive attitudes and action against illegal fishing.

Another key consideration is the differentiated nature of resource users. Although the poorest may be more dependent on natural resources, the wealthiest may be the heaviest extractors [90] and able to circumvent access restrictions [91]. As one interviewee pointed out, this creates a tension between strategies that will have the best outcomes for biodiversity and for poverty alleviation. Similarly, high natural resource dependency and lower social status for those in poverty restrict their ability to participate in poverty reduction programmes [81,92]. There are often larger forces at work in creating conservation problems at multiple organisational levels. Targeting only the livelihoods of local communities does not address wider drivers of unsustainable extraction such as fluctuating prices and political instability [90].

Livelihood-based interventions continue to attract significant donor funding [93]. While improving livelihoods is a good thing in its own right, and can foster improved relationships and trust between communities and conservationists [94], there is a lack of evidence that this will inevitably result in improved ecological outcomes. In designing these projects, there is a need to understand the drivers of unsustainable resource extraction, the livelihood profiles of communities and the priorities of resource users. In theory, projects that link livelihoods to biodiversity and local people and/or involve conditionality are more likely to succeed in terms of ecological outcomes, but this may involve trade-offs with poverty alleviation.

### Narrative 3: Compensation neutralises costs of conservation

This narrative accepts that there are unavoidable local costs to conservation in the form of access restrictions and human–wildlife conflict and assumes that these can be effectively offset thus fulfilling the ‘do no harm’ principle [95]. Compensatory approaches such as payments for harm caused by wildlife, resettlement, revenue sharing and development schemes, are driven not only by social justice concerns but also by efforts to reduce conflict and create positive attitudes towards conservation [96,97]. Increasingly, conservation is funded by major international donors who have explicit commitments to safeguard against negative social impacts and compensate for economic losses [98].

None of the reviewed literature was strongly supportive of this narrative, with only five papers providing some evidence that compensation is supported by local communities and at least partially offsets costs. The reasons related to both the compensation itself and the way in which schemes are implemented. First, the assumption that material compensation is commensurate with losses incurred from PAs is problematic. Compensation is often considered insufficient and not reflective of market values. In our sampled literature this was the case for compensation provided for a range of impacts including livestock loss [99,100], constraints on forest activities [101] and crop-raiding [102]. Material compensation is incommensurate with cultural losses. For example, although Twa communities received material benefits from revenue sharing from the Bwindi National Park, they have lost social freedoms and cultural heritage associated with hunting [103]. In Madagascar, many older households would be unwilling to stop the practice of swidden agriculture (*tavy*) in exchange for compensation, due to its socio-cultural value [104].

Material and monetary compensation is often provided for restricted access to land and displacement by PAs but may not account for material and non-material wellbeing losses. For example, land in resettlement villages was not perceived to be of comparable quality or quantity to that lost due to displacement from Suklaphanta Wildlife Reserve in Nepal, causing increased workloads, limited social interactions and reduced subjective wellbeing [105]. Land has cultural meaning, and places are intertwined with a sense of security, belonging, spirituality and identity that cannot be substituted [106,107]. Nevertheless, if community needs and aspirations are met, it is possible that resettlement can be carried out in a way that does not undermine people’s rights and wellbeing. For example, due to declining pastoral productivity and conflict with tigers, resettlement was the preferred option for the Gujjars in Nepal if it was associated with enhanced benefits including larger resettled land sizes, strengthened property rights and improved housing [108].

Although there was a mixture of views among our interviewees on the validity of this narrative, those that agreed were cautious in their support due to the difficulties in quantifying the meaning that livelihood practices hold, the practical challenges in administering compensation and unfulfilled promises made by government agencies. But several respondents explained how compensation can play an important role and provide a level of legitimacy for PA interventions, where there are tangible losses such as to livestock and agriculture. In a human–wildlife conflict compensation scheme in India, despite numerous shortcomings, respondents still supported a reformed compensation approach where conflict cannot be avoided [100]. The prevalent view amongst our respondents was that although not sufficient as a standalone approach, appropriate and timely compensation can be an important element of conservation if reinforced with greater engagement and recognition of costs. This should involve commitment that goes beyond the provision of one-off payments to include, for example, preventative measures to reduce human–wildlife conflict. However, two respondents raised the point that the whole idea of compensation removes power and incentives away from communities to manage ecosystems sustainably.

Even if compensation can work in theory, in practice schemes are often poorly implemented and administered. The process of claiming compensation can be long and tedious involving elaborate paperwork [99] and high transaction costs [100]. Where development projects are implemented, there can be a temporal mismatch whereby costs from resource access restrictions are immediate, but the benefits take time to emerge [101]. Limitations on the wildlife species included in compensation schemes or inappropriate methods to estimate compensation result in insufficient compensation [109]. Governments may fail to honour their commitments where compensation is not enshrined in policy or is associated with problems of corruption [100]. Inadequate or delayed compensation can develop deeply held grievances resulting in retaliatory killing of wildlife [110].

There is significant evidence of distributional inequity in compensation programmes. Development programmes may not reach those experiencing the greatest costs from PAs, but instead cluster around village and tourist centres, exacerbating economic inequalities [101,111]. There are often barriers to the most vulnerable groups accessing compensation. Households receiving compensation tend to be larger and wealthier [100], more food secure, better socially connected and live in more accessible areas [112]. Women and the poor face greater difficulty in accessing compensation as they lack official title to land, awareness of schemes, literacy, time and familiarity with bureaucratic procedures [100,105]. Even where monetary compensation reaches the poor, they may not have the capacity to reinvest in buying land and restoring livelihoods [113]. The result is that marginalised groups receive the least from compensation, if anything at all, even in cases where safeguarding procedures are in place to ensure the contrary [112].

In summary, the evidence rejects the idea that compensation as implemented is enough to substitute for experienced costs that often encompass non-material aspects of wellbeing and injustices. This does not mean that compensation is unnecessary, but it is rarely sufficient or commensurate. In addition, compensation mechanisms often do not work in practice, undermining social justice and support for conservation. Furthermore, our review suggests that there are situations in which compensation will never be commensurate with the loss incurred, thereby demanding greater openness to culturally appropriate alternatives.

### Narrative 4: Participation is good for conservation

There are two inter-linked reasons why participation is assumed to be instrumental to effective conservation. Firstly, participation can empower local communities to govern resources sustainably, an argument that owes much to research into governing commons [114] and the value of local knowledge [115]. Secondly, participation may motivate local support and stewardship by providing economic and non-economic benefits [33]. In other words, this narrative holds that participation can provide both the opportunity and the motive for communities to support conservation.

Such a narrative has ensured that participation became a central tenet of mainstream PA governance policy [16]. In international law it has also been clarified that procedural rights (access to information, participation in decision-making and access to justice) need to be respected in the designation and management of PAs [15]. The participation narrative has not gone completely unchallenged: a counter-narrative emerged around the turn of the century, questioning the effectiveness of participatory and community-based conservation [25]. In development studies some proclaimed participation a ‘new tyranny’ that served to reinforce unequal power relations and state control [116].

In our sample of 20 articles, 18 were judged to support the narrative although only three showed a strong link between participation and ecological outcomes. This was reflected by the interviews, where all respondents agreed with the narrative, except two who remained neutral. This body of research largely confirms that participation contributes to both motivation and capacity to support conservation, but also qualifies this in terms of the range of benefits that can motivate local people and the quality of participation that is required to empower people. Motivations for participation appear to vary across cases, and across different social groups. Participation can be motivated by expected livelihood benefits [117–119], but there are also several cases in which participatory conservation fails to deliver livelihood benefits yet is still valued for other reasons such as improved social capital [120], and sense of control [121]. According to one study, material motives are more important to men, whilst social motives are more important to women [122]. Whilst women may value participation for non-economic reasons, they are often less able to participate, due to constraints on their time or social barriers to taking on public roles [117,119,123–125]. As confirmed by interviewees, participation can thus impose a social cost due to lost time or livelihoods that outweigh the benefits of participating, so transaction costs need to be minimised. On balance, the evidence confirms that the opportunity to participate in PA management is widely valued by local communities.

The studies reviewed show us that the linkage between participation and effective conservation is not contingent on delivering livelihood benefits but can arise from either satisfying other needs and interests and/or triggering community capacity to control resource use. For example, a forest co-management programme in Malawi was found to have no short- or medium-term effect on household incomes, but participating households still cleared less forest than non-participants [126]. Similarly, participatory forest management in Tanzania did not provide measurable gains in wellbeing but forest governance was improved by reviving the community’s capacity to exclude outsiders [121]. Whilst community rights may be sufficient to unlock local capacity to manage resources, a study of marine fisheries in Kenya found that community co-management rights only led to positive ecological outcomes in conjunction with the establishment of no-take marine reserves highlighting the need for conducive socio-economic conditions and institutional capacities of communities [127].

The reviewed studies show that local contexts lead to variation in what motivates participation and what communities can achieve with rights to participate. One finding that is consistent across all of the studies and confirmed in interviews, is that the quality of participation is crucial in determining both motive and capacity for conservation [128]. Participation is often tokenistic and superficial, and this is recognised by communities as constraining what they can achieve. Interviewees highlighted that meaningful participation means having the power to effect change regarding ecosystem governance. In a survey in the Taita Hills, Kenya, 33% of respondents identified the superficiality of participation as the greatest constraint on forest conservation [122]. In both of the negative cases in our sample, the quality of participation is a key factor in undermining benefits to communities although there is evidence that ecological outcomes are positive at least in the short-term due to access restrictions [129,130]. Four papers that were categorised as partially supportive showed that superficial participation had negative implications for sustainability. In the study of WMAs in Tanzania, participation was manipulative, disempowering and went hand in hand with demonstrable harm to local livelihoods [129]. However, as highlighted in the interviews, participation is an evolving process, and one that needs sufficient time and resources to allow people to build relationships and negotiation skills, a point evidenced in the broader literature [131,132].

Our interviewees highlighted that the time, capacity and resources required for effective participatory processes often require the support of external agencies who can share the costs. For example, multi-community partnerships in marine PA sites in the Comoros, involving networks of communities, government and NGO actors, facilitated cooperation in fishery management ensuring all communities cooperated in fishery management on an equal footing [128]. Similarly, participation in marine PA sites in Indonesia was more extensive if management groups were supported by external institutions, such as through partnership with NGOs, academia and other community groups [133]. However, internal power structures will affect how participation takes place. Working through established customary governance arrangements is an effective route to establishing participatory conservation, but without mediation to steer negotiations towards inclusive governance, minority interests may get sidelined with repercussions for long-term sustainability [134].

In summary, this narrative is supported by our review, although there is limited evidence linking participation to ecological outcomes. Based on our findings we would qualify the narrative somewhat, such that participation supports PA effectiveness where it genuinely empowers communities and provides benefits that are locally valued and equitably distributed.

### Narrative 5: Secure tenure rights for local communities support effective conservation

Secure tenure rights are increasingly considered an important foundation for attaining positive conservation outcomes as they may increase the local legitimacy of and participation in conservation governance, promote the sustainable use of resources and foster local environmental stewardship against internal and external pressures [34,135]. The scope of legitimate tenure rights is not limited to individual property rights, which are often afforded greater legal status. Prominent theories, frameworks and international policy guidance defining tenure specifically include multiple types of tenure, and pivotally for conservation practice this includes customary and communal regimes and institutions [136], which are often side-lined as they comprise ‘informal arrangements’ and ‘unwritten customs and practices’ [137]. Increasing attention to security of tenure rights in conservation policy has resulted in the enhanced inclusion of areas managed by local communities within the global PA network [35]. Indigenous peoples already manage more than a quarter of the world’s land area but may struggle to protect these areas due to weak rights [138]. Clear and secure tenure rights are also pivotal for policy instruments such as PES or Reducing Emissions from Deforestation and forest Degradation (REDD+) to determine who is eligible to receive benefits and who is responsible for meeting contractual obligations [139]. Although absent in the Millennium Development Goals, tenure rights appear in five of the Sustainable Development Goals [140].

Of the 20 sampled articles addressing this narrative, none provide opposing evidence while 11 provide strongly supportive evidence. Six of those show evidence of a positive association whereby recognition of tenure rights leads to enhanced social and ecological outcomes, whereas five exhibit a negative association through which violation of or insecurity caused to local communities’ tenure rights through externally driven conservation interventions produces negative social and ecological outcomes. A further eight studies provide partial support for the narrative but assume the positive or negative social impacts promote or harm conservation, respectively, without providing specific evidence. The one remaining study suggests that secure individual property rights enhance conservation, although without paying any attention to other forms of tenure or potential social impacts of favouring a formal, individual tenure system [141]. Interviewees were also largely supportive of this narrative.

This set of cases highlights the pivotal importance of both tenure security based on customary and communal systems and of the scope of local influence in governance processes. At the most basic level, negative associations tend to occur when conservation interventions negate user rights with no regard for local needs or customary and communal institutions. In this situation, when a hegemonic model of conservation overrides existing systems through which rights are allocated among local communities, imposed structures may have negligible legitimacy and be entirely disregarded so that conservation goals are not met [142–144]. For example, where conservation interventions recognise only legal or individual property rights as compatible with conservation rules and override customary and communal local institutions, this may favour more powerful local or non-local actors to the detriment of vulnerable groups including the poor, women and cultural minorities. Those requiring access to support livelihoods or engage in cultural practices may act in defence of their needs and rights by seeking to establish an alternative to exclusive conservation rules, often through negotiation with alternate authorities such as sympathetic local government officials, as described by Rahman et al. [145] in Bangladesh. Such a situation can open the door to elite collusion and capture because the conservation intervention triggers a renegotiation of tenure rights, threatening ecological integrity both outside of and within PAs [146,147]. Instances of negative social and ecological outcomes resulting from imposed tenure regimes were also evidenced through contemporary governance approaches such as REDD+ [148,149].

The evidence suggests that to establish appropriate tenure security and sufficient rights to foster effective local stewardship, locally supported institutions that may have formed over long timescales need to be embedded within conservation structures that give sufficient confidence they will endure. Clear positive examples among the sample studies of recognising rights by embedding local tenure institutions within conservation included the Kasigau Corridor REDD+ project in Kenya that recognised communal forest tenure regimes [150], and the engaged stewardship and mobilisation of resistance to unsustainable logging in Cambodia [151]. Where secure tenure supports local livelihoods and fosters effective local stewardship it can be particularly important to protect those governance structures. For example, weakening of rights for betel nut growers in Soppinabetta forests in the Western Ghats of India to control resource use on their land led to many selling it for extractive development [152].

It is also important to consider the extent of rights granted to different groups of people, looking beyond basic user rights to address rights of control and authority that determine who has power to make decisions about resource allocation and influence governance structures [153]. Provision of use rights alone may not be enough to prevent tenure insecurity arising, because if people fear those rights are likely to be removed or overruled and they lack any power to block those decisions, positive feedbacks for conservation may be foregone, as exemplified by Davis [154] for Maasai pastoralists impacted by a WMA in Tanzania. Indeed, three interviewees highlighted difficulties for pastoralist groups whose dynamic and seasonal customary systems of land and resource rights may be threatened through tenure formalisation processes. To nuance these findings further, several studies highlight the dangers of romanticising local institutions and their ability to govern natural resources adaptively and inclusively, particularly because management capacity and local cohesion may be lacking in the face of numerous drivers of social and institutional change at the local level [144,155].

Our review also revealed the need to explore not just tenure systems but perceptions about the security of tenure rights, which can be a key determinant of behavioural change, even when tenure arrangements appear stable. Local perceptions of tenure security can be highly influenced by past experiences of policies enacted by states or colonial powers, and conservation interventions can be perceived as extensions of them [156,157]. Even if conservation authorities are trusted, perceived tenure security may be weak where the central government has a record of overriding them, for example, through the proliferation of land concessions for commercial, infrastructure and extractive industries in Cambodia [151].

In summary, secure tenure rights can empower communities to sustainably manage resources and participate in effective ecosystem governance. However, respect for customary and communal access systems, and trust in the governance arrangements are critical for success.

## Discussion

Our review shows that, in their simplest forms, commonly employed narratives linking protected areas to human wellbeing are not borne out in practice and a range of factors add complexity to the narratives. Crucially, our review illustrates that the model of conservation that is legitimated by simplistic versions of these narratives can inhibit the attainment of both the wellbeing of Indigenous Peoples and Local Communities and, ultimately, effective nature conservation. The findings suggest those involved in conservation need to critically examine the political nature of the ideas they adhere to, the way they are used to justify interventions and their means of implementation and serve to obscure local voices and experiences. Reductionist approaches to poverty alleviation, participation, benefit sharing and tenure all fall short of supporting rights, avoiding harms and in many cases of producing positive social and ecological outcomes. Our review findings are in line with a transformation towards decolonised and justice centred forms of conservation [158,159] and highlight ways in which the post-2020 biodiversity conservation strategies need to more explicitly and proactively integrate social equity, restorative justice, human rights and appreciate the social–cultural contexts and political histories of PA sites. We note points of progress in addressing social equity concerns in the draft of the post-2020 Global Biodiversity Framework [3], but the findings from our evidence synthesis also highlight significant gaps that continue to impede progress towards more equitable conservation that respects the rights of Indigenous Peoples and Local Communities in practice. Below we suggest how the 21 targets could be enhanced as well as interpreted and implemented at national and local levels (Table 2).

**Table 2.** Implications of the narrative evidence review for the application of the post-2020 Global Biodiversity Framework

Narrative shorthand	Implication of the review for conservation practice and the application of post-2020 CBD targets	Related goals and targets in draft post-2020 Global Biodiversity Framework [3]
N1: Conservation is pro-poor	The full range of material and non-material costs and benefits PAs can have for local communities should be explicitly acknowledged, identified, assessed and addressed for conservation governance of any form Ensure rights of access to local communities for sustainable resource use and cultural practices (see also narrative 5) Proactive measures to ensure the poor and marginalised are represented and access benefits (e.g., redistribution of tourism income) Restorative justice approaches to agree appropriate ways to redress historical and continuing harms	Ensure benefits, especially for the most vulnerable Integrate biodiversity into poverty reduction strategies Respect rights of IPLCs over resources
N2: Poverty reduction benefits conservation	Poverty reduction strategies must consider local definitions of poverty and deprivation beyond income Role of biodiversity in subsistence and meeting basic needs to be valued and safeguarded Any benefits should be culturally appropriate Interventions and programmes should aim to support local institutions and practices, not replace them	Ensure benefits, especially for the most vulnerable Sustainable management of production systems Benefit sharing from traditional knowledge Integrate biodiversity into poverty reduction strategies Ensure traditional knowledge guides decision-making
N3: Compensation neutralises costs of conservation	Harms should be a last resort due to difficulties in making compensation fair or commensurate in practice Where harms are unavoidable, ensure compensation attends to non-material and cultural losses as well as economic losses Compensation schemes require equitable governance in the long-term, as benefits achieve little without empowerment and respect for local knowledge and institutions Specific attention to the poorest, most marginal groups including women because elite capture should be expected	Ensure benefits, especially for the most vulnerable Benefit sharing Reform harmful incentives
N4: Local participation is good for conservation	Focus on the extent and quality of participation (or of governance more broadly) rather than its occurrence Establish and uphold standards for the continual influence and control of Iocal communities, from design stages, and a central role for local knowledge and institutions in governance Decision making through locally legitimate authority, maximising inclusion especially for women Establish relationships, trust between communities and non-local organisations, through conflict resolution as precursor to decision-making where necessary	Ensure traditional knowledge guides decision-making Equitable participation in decision-making Integrate biodiversity into poverty reduction strategies
N5: Secure tenure rights support effective conservation	Define tenure to include customary and communal aspects, beyond individual, legal property rights All signatory nations to CBD and authorities for any conservation programme should report on the assessment and inclusion of Indigenous Peoples and Local Communities’ communal and customary tenure systems Establishment of new PAs or restoration programmes should build upon local traditional knowledge and institutions, and legitimise and support local tenure systems Particular attention required to include the poor and marginalised social groups for whom land and resource access can be temporary, rented and undocumented tenancy	Targets for restoration and PA area extent Ensure benefits, especially for the most vulnerable Sustainable management of production systems Nature-based solutions Integrate biodiversity into poverty reduction strategies Reform harmful incentives Ensure traditional knowledge guides decision-making Respect rights of IPLCs over resources

Our analysis was based on a relatively small number of papers, and these were biased towards certain regions, and are certainly not representative of all PAs in the Global South. The studies also capture likely publishing bias against results of no impact. We counterbalanced this bias through interviews with experts with a variety of perspectives and experiences relating to PAs around the world including in the Americas. However, further research would be needed to discern how the narratives may play out differently in Latin America which is underrepresented in our study. Overall, our aim was to focus less on how common certain outcomes are but on how the narratives are complicated by realities to provide insights into how the relationships between PAs and wellbeing can be strengthened. We also recognise that there are other narratives underpinning conservation practice. The five we selected were deemed to be common and fundamental to interventions, but others are likely to exist, and likewise need to be critically examined.

The simple assumption of N1 that ‘conservation is pro-poor’ can be mis-used to legitimise exclusionary PAs and systems of governance that are too often harmful for the wellbeing of communities. The pro-poor narrative is bolstered by the assumption that any costs to the poor can be suitably compensated for (N3). The counterclaim found in our review is that if conservation is to be genuinely pro-poor it will need to embrace a model that prevents harms rather than seeking to compensate for them. Instead any human rights restriction arising from PAs and subsequent compensation should be seen as a last resort. We also found N2 ‘poverty reduction benefits conservation’ to be a problematic narrative, in particular where this assumes that efforts to support livelihoods will lead to conservation effectiveness.

There was more support for narratives 4 and 5 on participation and secure tenure rights, respectively, especially among our interviewees, pointing to the redistribution of power towards communities as important for conservation success over improvements and compensation in material poverty on their own. Although conservation can succeed in its ecological aims through enforcement [160] and participatory arrangements are far from being a panacea [161], the ethical basis for ensuring equity in conservation is well-accepted [16]. Recent research outside our sample tends to confirm that participation by local people can help to deliver both ecological and social objectives of PAs [21,162–165]. It is striking, however, that even in so-called participatory forms of governance and tenure reform there is a tendency for elite capture and costs for the most marginalised. This highlights the vital importance of meaningful participation that genuinely empowers people to effect change through iterative and culturally appropriate processes, with benefits being distributed equitably, and the recognition of customary tenure rights that give authority and control to communities [166].

Our review of evidence urges caution about the proposed expansion of PAs under the current draft of the post-2020 framework. At a superficial level, the 21 draft targets [3] appear to cover the multiple dimensions of equity or justice (distribution of costs and benefits, decision-making procedures and recognition of values, knowledge systems and institutions), through which the concerns of Indigenous Peoples and Local Communities and the poorest among them are often articulated [24]. The targets go slightly beyond previous principles by stating that systems of customary sustainable use should be protected (Target 9), and that communities, especially the most vulnerable, should receive equitable benefits from conservation, including nutrition, food security, medicines and livelihoods (Targets 9 and 13). Equitable and effective participation in decision-making and free prior and informed consent are explicitly targeted (Targets 13, 20 and 21), while respect for traditional knowledge and practices (Targets 13 and 20) and rights over land, territories and resources, for Indigenous Peoples and Local Communities as well as women, girls and youth (Target 21) appear to also be safeguarded [3]. Yet gaps remain between those principles and the nuanced issues highlighted through our evidence review (see Table 2). In general, the targets highlight Indigenous Peoples and Local Communities, women, youth and the vulnerable primarily as potentially impacted parties and a group of actors to be considered stakeholders, whereas phrasing should more proactively endorse their empowerment in PA governance and recognise the essential role their cultural values, customary institutions and stewardship actions play in conservation. Proposed targets do enshrine the importance of local community participation, yet ensuring the quality of participation remains the challenge. Genuine and enforceable procedural standards are needed, informed by the understanding that participation is an iterative process requiring time, resource, mutual learning, trust-building and respect for local forms of knowledge and decision-making [14].

To foster meaningful inclusion and empowerment, attention must be specifically directed to the past experiences of displacement, disruption of knowledge systems and cultural practices, and political marginalisation suffered by many through colonisation, market-driven development and previous conservation interventions, which influence current relationships, expectations and the implementation of any current or future conservation initiative [11]. This has profound implications for the processes required to build the requisite trust for inclusion of the most vulnerable and marginalised, and to develop intercultural understanding for collaboration between plural knowledge systems. Such processes may entail conflict resolution or restorative justice approaches to attend to any historical and continuing effects on people’s wellbeing, their institutions, tenure security and rights, relationships and agency [116,129].

In reality, while principles of equity have been espoused in global environmental agreements for at least 20 years [167], many national legal and political frameworks simply do not support the rights, cultural practices and institutions or empowered political influence of Indigenous Peoples and Local Communities, and consequently neither do many conservation interventions [159]. This persistent barrier to equitable conservation in practice means that the Global Biodiversity Framework must look beyond the principles themselves to focus more attention to the way those social and governance standards are to be implemented. Of importance here is the cursory reference under section J paragraph 18 to ‘responsibility and transparency…… in implementation of the framework’ [3]. The approaches for ensuring rights of access and tenure, territorial integrity and equitable and effective participation have seldom been monitored, reported or reviewed at any level in the past, resulting in an absence of accountability if social standards are not met [168]. These governance issues and the pathways to address them should be explicitly articulated. Governance quality, particularly an emerging focus on equity and rights, is receiving increasing attention within conservation policies [167], multi-stakeholder processes [169] and assessment tools [170], with potential to expose the flaws of conservation based on external assumptions about local communities and promote more nuanced approaches.

Our research does not suggest that we should abandon attempts to link improvements in biodiversity and human wellbeing, but highlights the need for certain governance qualities, such as inclusiveness and adaptability. Dynamics for a given location fundamentally shape the relationships posited in the narratives, undermining the application of any kind of blue-print model for successful conservation and assumed synergies with local wellbeing, regardless of context. Conservation policy and practice therefore needs to reorient towards theories of change and types of governance more integrally structured around local knowledge and perspectives [171]. At the same time, conservationists need to recognise that communities invariably embody power dynamics allowing the well-placed to benefit from any intervention or change at the expense of the less well-placed. Women in particular tend to lose out in conservation processes, and gendered approaches to governance and impact evaluation are needed [172,173].

Evidence across all the narratives reviewed highlights the importance of understanding wellbeing from the ground up rather than assuming people’s priorities and motivations [174]. This understanding must go beyond material dimensions to account for aspects of people’s lives that they value, and extend to ideas of justice, culturally specific relations with nature, customary tenure regimes and livelihoods. The evidence suggests that despite qualitative data on perceptions often being dismissed as ‘unscientific’ in the conservation literature [175], understanding local values and viewpoints such as perceived tenure security is vital in creating synergies between ecological and social outcomes. All too often, impact assessments of conservation focus on financial and material outcomes to the exclusion of social and cultural impacts [176]. Lack of attention to local values partly explains unfulfilled expectations, poor motivation and lack of local legitimacy, a thread running through the evidence base. For example, compensation should include consideration of immaterial damage affecting Indigenous Peoples and Local Communities’ subsistence and spiritual connection with their territory [177].

The packaging of PAs as win–wins for biodiversity and human wellbeing downplays the inevitable trade-offs that occur in conservation and highlighted by our review between social and ecological outcomes, aspects of wellbeing, groups of people and different scales [178]. Acknowledgement of trade-offs supports more realistic acceptance of losses and opens up negotiation over choices and novel ideas about what success means and how to reduce or eliminate trade-offs, or what may not be appropriate to ‘trade-off’ [179,180]. In achieving the proposal to integrate biodiversity values into planning and development processes, governance structures must allow local participation in deliberations over wellbeing priorities, how they may link to biodiversity and the ecological realm, with recognition given to place-based knowledge about nature [181].

Our review also highlights the value of taking a broader perspective beyond the boundaries of PAs, local communities and the present. Broader structural issues such as non-local resource demand and government policies are often the underlying cause of overexploitation of resources, poverty and changes in local management institutions and values [182,183]. Perhaps because of the difficulties of challenging these issues, conservationists have long focused on local ‘threats’ and individual agency [184]. Social justice approaches make imperative the need to shape broader drivers, requiring political engagement at multiple scales of governance on longer timescales. For example, historical injustices and land tenure policies that create insecurity must be redressed to build trust in current projects. Likewise, people’s priorities and conceptions of wellbeing will change within dynamic systems that shape people’s needs and desires, necessitating both adaptive governance systems and attention to the shifting broader socio-economic and political factors that may influence unsustainable practices.

The conservation community have increasingly acknowledged the importance of considering local peoples’ experiences of and agency in conservation. But current proposals for meeting ambitious targets for protection post-2020 (e.g., [185,186]) need greater clarity on key issues such as governance qualities and how costs to local communities should ideally be mitigated, if unavoidable. The focus within global biodiversity policy debates on what proportion of the Earth to conserve, rather than how it is to be conserved, threatens to downplay the importance of addressing deficiencies in governance and equity outcomes from existing PAs as well as the broader drivers of unsustainable resource extraction. Our review suggests that future approaches should draw upon just and democratic forms of conservation that put local actors at the centre of decision-making and recognise their rights to land and resources and ensure conservation actors are accountable for upholding governance and equity standards. However, the lessons from 15 years of literature exploring the relationships between local people and protected areas and the experiences of practitioners highlights the complex building of collaboration and progressive political change this requires.

## Figures and Tables

**Table:** 

Organisation	Narrative				
	1	2	3	4	5
A Rocha*					
Africa Conservation Fund UK					
African Conservation Centre					
African Ele-Fund					
African Fund for Endangered Wildlife					
African Mangrove Network (AMN)					
African Parks Foundation					
African Wildlife Foundation					
Amara Conservation					
Ambassadors for Wildlife through Education (AWE)					
Amboseli Community Wildlife Tourism Project (ACWTP)					
Amboseli Trust for Elephants					
Ape Action Africa					
Arboretum D’Antsokay					
ARCOS – Albertine Rift Conservation Society					
Association ‘Les Amis des Oiseaux’ (AAO)					
Association pour la Conservation de la Nature au Rwanda					
Biodiversity Foundation for Africa (BFA)					
BirdLife Botswana (BLB)					
Birdlife International (Secretariat)*					
BirdLife Zimbabwe (BLZ)					
Bonobo Conservation Initiative (BCI)					
Born Free Foundation*					
Bushmeat Project					
Cameroon Environmental Watch					
Caracal					
CERCOPAN					
Cheetah Conservation Botswana					
Cheetah Conservation Fund					
Chobe Wildlife Trust					
Conservation International*					
Conservation Society of Sierra Leone (CSSL)					
Conservation South Luangwa					
Conservation through Poverty Alleviation					
Conserve Africa					
David Sheldrick Wildlife Trust					
David Shepherd Wildlife Foundation*					
Dian Fossey Gorilla Fund International					
Durrell Wildlife Conservation Trust*					
Earthwatch Institute*					
East African Wild Life Society					
Elephant Pepper Development Trust					
Elephant Voices					
Endangered Wildlife Trust (EWT)					
Environmental Foundation for Africa, Sierra Leone					
Ethiopian Wolf Conservation Programme					
Fanamby					
Fauna and Flora International*					
Fondation Pour les Aires Protegées at la Biodiversite de Madagascar					
Fondation Tany Meva					
Frankfurt Zoological Society*					
Friedkin Conservation Fund					
Friends of Conservation (UK)					
Friends of Conservation (USA)					
Friends of Elephant/Vrienden van de Olifant					
Friends of the Hippo and The Turgwe Hippo Trust					
Gallmann Memorial Foundation (GMF)					
George Adamson Wildlife Preservation Trust					
Ghana Wildlife Society (GWS)					
GORILLA Association					
Gorilla Organization					
Grassland Society of Southern Africa					
H.E.L.P. Congo					
Humane Society International*					
Hurghada Environmental Protection & Conservation Association					
International Elephant Foundation*					
International Rhino Foundation (IRF)*					
Jane Goodall Institute					
Kasanka Trust					
Kenya Wildlife Trust					
Kilimanjaro Environmental Conservation Management Trust Fund					
Kipepeo Butterfly Project					
Laikipia Wildlife Forum					
Last Great Ape Organization					
Lewa Wildlife Conservancy					
Lion Conservation Fund					
Living with Lions					
Lukuru Wildlife Research Project (LWRP)					
Maasai Foundation of East Africa					
Madagascar Fauna Group					
Madagascar Wildlife Conservation					
Man And the Environment (MATE)					
Mokolodi Wildlife Foundation					
Mount Kenya Wildlife Conservancy					
Mpala Wildlife Foundation					
Naturama					
Nature Djibouti					
Nature Kenya: The East Africa Natural History Society					
Nature Seychelles					
NatureUganda (NU)					
Nigerian Conservation Foundation (NCF)					
Nigerian Montane Forests Project					
Noé Conservation					
Northern Rangelands Trust					
Nouvelles Approches (now Biodiversité au Katanga)					
Organização para a Defesa eDesenvolvimento das Zonas Húmidas					
Owens Foundation for Wildlife Conservation					
Painted Dog Conservation Trust					
Pan African Sanctuary Alliance (PASA)					
Pandrillus					
Partners in Conservation					
Peace Parks Foundation					
Peregrine Fund*					
Predator Conservation Trust					
Project Primate					
Prowildlife					
Rainforest Action Network (RAN)*					
Rainforest Foundation*					
Rare Species Conservatory Foundation*					
Rhino Ark					
Robin Hurt Wildlife Foundation					
RSPB International*					
Safari Club International Foundation*					
Sahara Conservation Fund					
Save My Future Foundation (SAMFU)					
Save The Elephants (STE)*					
Save the Rhino International*					
Save the Rhino Trust					
Save the Species Worldwide Foundation*					
Sebakwe Black Rhino Trust					
SEED Madagascar					
Somali Environmental Protection and Anti-Desertification Organisation					
Southern African Wildlife College					
Space for Elephants					
System of Protected Areas of Madagascar (SPAM)					
Tandroy Conservation Trust					
Tanzania Forest Conservation Group					
Tanzania Natural Resources Forum					
The Colobus Trust					
The International Crane Foundation*					
The Kesho Trust					
The Rainforest Foundation Fund Inc (Norway)					
The Wasmoeth Wildlife Foundation					
Trees for the Future					
TUSK Trust					
Uganda Conservation Foundation					
West African Primate Conservation Action (WAPCA)					
West African Bird Studies Association (WABSA)					
West Lunga Trust					
Westerveld Conservation Trust					
Wetlands International*					
Wild Chimpanzee Foundation					
WILD Foundation*					
Wilderness Trust of Southern Africa					
Wildlife Action Group Malawi					
Wildlife and Environment Society of Malawi					
Wildlife Conservation Foundation of Tanzania					
Wildlife Conservation Society (WCS)*					
Wildlife Conservation Society of Tanzania (WCST)					
Wildlife Direct					
Wildlife Now					
Wildlife Warriors Worldwide*					
WildTrack*					
William Holden Wildlife Foundation					
World Parrot Trust*					
World Resources Institute*					
World Turtle Trust*					
WWF (International)*					
WWF UK					
WWF US					
Zambezi Society					
Zimbabwe Conservation Task Force					
Zoological Society for the Conservation of Species and Populations					
Zoological Society of Milwaukee					
	118	108	53	84	39

**Table:** 

Country	World Bank Economic category	Region (United Nations geoscheme)
Afghanistan	Low income	Asia
Armenia	Lower middle income	Asia
Bangladesh	Lower middle income	Asia
Benin	Low income	Africa
Bhutan	Lower middle income	Asia
Bolivia	Lower middle income	Americas
Burkina Faso	Low income	Africa
Burundi	Low income	Africa
Cabo Verde	Lower middle income	Africa
Cambodia	Lower middle income	Asia
Cameroon	Lower middle income	Africa
Central African Republic	Low income	Africa
Chad	Low income	Africa
Comoros	Low income	Africa
Congo, Dem. Rep.	Low income	Africa
Congo, Rep.	Lower middle income	Africa
Côte d'Ivoire	Lower middle income	Africa
Djibouti	Lower middle income	Africa
Egypt, Arab Rep.	Lower middle income	Africa
El Salvador	Lower middle income	Americas
Eritrea	Low income	Africa
Ethiopia	Low income	Africa
Gambia, The	Low income	Africa
Ghana	Lower middle income	Africa
Guatemala	Lower middle income	Americas
Guinea	Low income	Africa
Guinea-Bissau	Low income	Africa
Haiti	Low income	Americas
Honduras	Lower middle income	Americas
India	Lower middle income	Asia
Indonesia	Lower middle income	Asia
Kenya	Lower middle income	Africa
Kiribati	Lower middle income	Oceania
Korea, Dem. People's Rep.	Low income	Asia
Kosovo	Lower middle income	Europe
Kyrgyz Republic	Lower middle income	Asia
Lao PDR	Lower middle income	Asia
Lesotho	Lower middle income	Africa
Liberia	Low income	Africa
Madagascar	Low income	Africa
Malawi	Low income	Africa
Mali	Low income	Africa
Mauritania	Lower middle income	Africa
Micronesia, Fed. Sts.	Lower middle income	Oceania
Moldova	Lower middle income	Europe
Mongolia	Lower middle income	Asia
Morocco	Lower middle income	Africa
Mozambique	Low income	Africa
Myanmar	Lower middle income	Asia
Nepal	Low income	Asia
Nicaragua	Lower middle income	Americas
Niger	Low income	Africa
Nigeria	Lower middle income	Africa
Pakistan	Lower middle income	Asia
Papua New Guinea	Lower middle income	Oceania
Philippines	Lower middle income	Asia
Rwanda	Low income	Africa
Samoa	Lower middle income	Oceania
São Tomé and Principe	Lower middle income	Africa
Senegal	Low income	Africa
Sierra Leone	Low income	Africa
Solomon Islands	Lower middle income	Oceania
Somalia	Low income	Africa
South Sudan	Low income	Africa
Sri Lanka	Lower middle income	Asia
Sudan	Lower middle income	Africa
Swaziland	Lower middle income	Africa
Syrian Arab Republic	Lower middle income	Asia
Tajikistan	Lower middle income	Asia
Tanzania	Low income	Africa
Timor-Leste	Lower middle income	Asia
Togo	Low income	Africa
Tonga	Lower middle income	Oceania
Tunisia	Lower middle income	Africa
Uganda	Low income	Africa
Ukraine	Lower middle income	Europe
Uzbekistan	Lower middle income	Asia
Vanuatu	Lower middle income	Oceania
Vietnam	Lower middle income	Asia
West Bank and Gaza	Lower middle income	Asia
Yemen, Rep.	Lower middle income	Asia
Zambia	Lower middle income	Africa
Zimbabwe	Low income	Africa

**Table:** 

*Bibliographic info*	Author/s	
	Year	
	Type of Biome	Terrestrial, marine
	Title	
	Affiliations of authors	Academic, independent (no affiliation), PA management, public sector, private sector, research institute
*Protected area (PA)*	Name of PA	
	IUCN Protected Area Category	Ia strict nature reserve; Ib wilderness area; II national park; III natural monument/feature; IV habitat/species management area; V protected landscape/seascape; VI protected area with sustainable use; buffer/transition zone; biosphere reserve; ICCA (not covered by IUCN); private (not covered by IUCN); other – specify, e.g., de facto, voluntary conservation; not reported
	Governance type/implementer	government, shared (diverse stakeholders), private, indigenous or local communities, not specified
	Country of PA	
	Year established (if stated)	
*Study info*	Year of study (if stated)	
	Data source	Primary, secondary
	Unit of analysis	individual, household, village, country
	Data type	Quantitative, qualitative, mixed
	Includes subjective measures	Y/N
	Outcomes reported on	Social, social and environmental
	Evidence of process	Outcomes reported only, process and outcomes reported, other
	Aspect of PA studied	establishment (presence/absence), management activities, governance processes, indirect (e.g., tourism or PES)
*Outcomes*	Social outcome/s reported	income, other material (food, assets, livelihoods, access to services), health, security, social relations/conflict, agency/empowerment/participation, cultural values/practices, subjective (overall, e.g., how's life? or satisfaction with specific aspects of wellbeing)
	Details of main outcomes covered	
	Wellbeing explicitly mentioned	Y/N
	Equity/distributional aspects of outcomes [differential outcomes reported]	Y/N
	Equity/distributional aspects of outcomes [equity explicitly mentioned]	Y/N
	Environmental outcomes or behaviours included [environmental outcomes (e.g., biodiversity)]	Y/N
	Environmental outcomes or behaviours included [Environmental behaviours (e.g., fishing, hunting)]	Y/N
	Environmental outcomes linked to social outcomes or processes	Y/N
	ES specified	None, supporting, provisioning, regulation, cultural
	Positive or negative social impacts	Positive, negative, mixed, not explicit
*Narratives*	1. Conservation is pro-poor [link to narrative]	Yes, no, partially
	1. Conservation is pro-poor [supportive of narrative]	Yes, no, partially
	1. Pro-poor narrative notes	
	2. Poverty reduction benefits conservation narrative [link to narrative]	Yes, no, partially
	2. Poverty reduction benefits conservation narrative [supportive of narrative]	Yes, no, partially
	2. Poverty reduction narrative notes	
	3. Compensation neutralises conservation costs [link to narrative]	Yes, no, partially
	3. Compensation neutralises conservation costs [supportive of narrative]	Yes, no, partially
	3. Compensation narrative notes	
	4. Participation is good for conservation [link to narrative]	Yes, no, partially
	4. Participation is good for conservation [supportive of narrative]	Yes, no, partially
	4. Participation narrative notes	
	5. Resource tenure underpins long-term conservation [link to narrative]	Yes, no, partially
	5. Resource tenure underpins long-term conservation [supportive of narrative]	

**Table:** 

Type of organisation	Based in	Region/country specialism
Conservation membership organisation	Kenya	Kenya
Country office of international conservation NGO	Laos	Lao
Country office of international conservation NGO	Nepal	Nepal
Country office of international conservation NGO	Pakistan	Pakistan
Government conservation agency	Uganda	Uganda
Government research institute	Kenya	Kenya
Grassroots development NGO	Kenya	Kenya
In-country conservation NGO	Madagascar	Madagascar
In-country conservation NGO	Brazil	Brazil
Intergovernmental organisation	Italy	Oceania
International conservation NGO	USA	Central Africa, Latin America and Asia
International conservation NGO	USA	Nepal, East and Southern Africa
International conservation NGO	USA	Colombia, Peru, Madagascar
International conservation policy organisation	UK	Africa
International development organisation	Netherlands	Latin America
NGO focused on indigenous rights	across Africa	South Africa
Research organisation on environment and development	UK	East Africa, West Africa

## Data Availability

The datasets generated during and/or analysed during the current study are available in the repository: https://doi.org/10.5522/04/17153291.

## Supporting information

### Conservation organisations

Evidence for narratives on conservation organisation websites (January 2018)

*Organisation operates internationally outside of Africa.

  Strongly evident    Weakly evident

### Interview validation

**World Bank Economies**

(March 2017)

### Exclusion Criteria

**Exclude on date:** The study has a publication date before 2014.

**Exclude on country:** The study is outside of the list of included countries for the IMPACTS project, which focuses on low- and lower middle-income countries only (according to the World Bank).

**Exclude on population**: The subjects of the intervention are not discrete individuals, households, communities or national states.

**Exclude on intervention**: The study does not include the establishment or management of an area-based protected or conserved area intervention or associated policy or programme.

**Exclude on outcome:** The study does not observe, measure or describe human wellbeing indicators, outcomes, or impacts. The study only focuses on biophysical outcomes of conservation or solely examines how status or trends in human wellbeing affect conservation outcomes.

**Exclude on study type:** The study is a theoretical or conceptual article, modelling study, commentary, editorial or narrative review.

### Search Terms

**Protected area intervention terms**

protected area* OR nature reserve* OR wilderness area* OR national park* OR natural monument* OR natural feature* OR management area* OR world heritage site* OR biosphere reserve* OR biodiversity conservation OR national reserve* OR conservanc* OR ecotourism OR corridor* OR community-based conservation OR payment for ecosystem services OR PES AND

**Intervention adjacent terms**

marine OR freshwater OR coastal OR forest* OR ecosystem* OR species OR habitat* OR biodiversity OR sustainab* OR ecolog* OR integrated OR landscape OR seascape OR coral reef* OR natural resource* AND

**Outcome terms**

wellbeing OR well-being OR well being OR ecosystem service* OR nutrition OR skill* OR empower* OR clean water OR livelihood* OR (food) security OR resilience* OR vulnerability OR (social) capital OR attitude* OR perception* OR (human) health* OR human capital OR (traditional knowledge or TEK) AND

**Outcome adjacent terms**

human* OR people OR person* OR community* OR household* OR fisher* OR collaborative

### Interview Questions

Please consider the following context and the widespread assumptions that follow. We would then like to ask about your experience, and your opinion on those assumptions, through a set of structured questions.

**STUDY CONTEXT**

- We are interested in understanding the processes involved in positive and negative social impacts of PAs on human wellbeing, how impacts may differ between groups, for example, according to gender and age, and how relationships between social and environmental outcomes may vary with context such as with governance type, economic and social factors.
- We are interested in impacts on *multi-dimensional* human wellbeing and equity. Therefore, please consider the following aspects:
  - Material wellbeing including assets, income, food, livelihoods and subsistence activities
  - Health
  - Security – confidence in the future
  - Social relations, for example, within the community and between communities and PA authorities
  - Agency, empowerment and participation
  - Recognition of and impacts on cultural values and practices
  - Subjective wellbeing – how people *feel* about change
- By ‘protected areas’, we mean all kinds of protected and conserved areas in marine and terrestrial habitats. This includes the six IUCN Protected Area management categories, plus other effective area-based conservation measures, such as other types of indigenous, community and privately conserved areas; and areas that provide connectivity between designated protected areas. We are interested in the establishment, management processes and activities associated with these areas.
- Our focus is on PAs and communities in low- and lower middle-income countries (according to the World Bank), the countries that are the target of ESPA work.

We are structuring the analysis by investigating five key narratives in the data, which we have identified as underlying PA establishment and management actions. We are interested in whether these assumptions hold in real-life situations.

**NARRATIVES**

**1. Ecosystem services narrative**

*Because poor people are disproportionately dependent on ecosystem services, protected areas that target those services will be pro-poor*

This narrative sees PAs as a tool to reduce poverty. It is often the poorest people who are most dependent on ecosystems for their livelihoods and food security. By protecting these ES, PAs are thus important for delivering poverty reduction objectives by supporting a range of economic activities such as forestry, fisheries, agriculture and tourism as well as providing access to clean water and energy.

**2. Poverty and conservation narrative**

*Because poor people are disproportionately dependent on ecosystem services, improving their material wellbeing will reduce pressure on protected areas*

This narrative sees poverty reduction as a means to achieve PA conservation. This can occur through strategies such as alternative livelihoods, revenue sharing, investment in infrastructure and tourism, which are implemented to reduce reliance on natural resources and behaviours that might be environmentally damaging. The economic benefits generated by these strategies also increase local support for conservation. Incentive schemes such as PES that provide benefits to poor people are directly linked to PA conservation objectives, thus enhancing conservation success.

**3. Compensation narrative**

*Unavoidable social costs of protected areas for poor people can be neutralised by providing appropriate compensation*

Because poor people endure the costs of PAs, such as due to human–wildlife conflict (crop-raiding, livestock predation) or reduced access to PA resources for food, fuel or livelihoods, compensation schemes offset these costs and create more positive attitudes towards PAs.

**4. Participation narrative**

*Participation in protected area governance is seen by communities as a positive social benefit and it is a route to effective conservation*

This narrative sees participation of local people in PA decision-making and recognition of their values and interests as important factors contributing to long-term conservation success. Participation is valued by communities and increases the legitimacy of the PA and its policies. Participation increases communities’ sense of stewardship over PAs, builds capacity and creates greater incentives to harvest resources sustainably to ensure future access to benefits.

**5. Secure tenure narrative**

*Secure land and resource tenure underpins improved conservation outcomes (social and ecological) in and around protected areas*

This assumes that tenure insecurity is the cause of resource degradation. Secure land rights enable poor people to invest in resources and strategies that promote resilient livelihoods. It is also the foundation for the sustainable use of resources that supports long-term conservation outcomes.

We would like to ask you the following questions, and for you to share your thoughts based on your own experiences through your work on ESPA projects or other similar projects or research.

Overall:

1. From your ESPA project/other professional experience, can you give examples of a) positive and b) negative social impacts of PAs on the wellbeing of local people? Can you explain why these might have occurred? Where they have been negative? What has been done to reduce them?
2. From your own experience, what are the *synergies* you’ve found in PA conservation (i.e., different outcomes interact to improve the overall outcome) a) between social and ecological outcomes, and b) between different social outcomes? Can you give examples of each from your work?
3. What do you see as some of the critical trade-offs in protected area conservation, a) between social and ecological outcomes, and b) between different social outcomes? Can you give examples of each from your work? Are there difficult decisions that you’ve had to make, or you have seen others make, in regard to these trade-offs, and how they have been resolved?

Protected area narratives

For each of the narratives above can you/answer the following:

1. Is this assumption familiar to you? (circle as appropriate)
2. Where have you come across the idea?
3. Do you think the assumption has changed through time? How?
4. From your experience do you agree that the assumption is valid? (circle as appropriate)
5. If at all valid, can you provide examples? Where? In what kinds of PA? For whom?
6. If you do not think it is completely valid in what ways or in which contexts do you think it is not valid? Where and for whom? Please refer to specific examples such as case studies or papers.
7. Do you have an additional narrative or idea that you think guides PA management?
